# Remineralization Strategies for Teeth with Molar Incisor Hypomineralization (MIH): A Literature Review

**DOI:** 10.3390/dj11030080

**Published:** 2023-03-13

**Authors:** Joachim Enax, Bennett T. Amaechi, Rayane Farah, Jungyi Alexis Liu, Erik Schulze zur Wiesche, Frederic Meyer

**Affiliations:** 1 Research Department, Dr. Kurt Wolff GmbH & Co. KG, Johanneswerkstr. 34-36, 33611 Bielefeld, Germany; 2 Department of Comprehensive Dentistry, School of Dentistry, University of Texas Health San Antonio, 7703 Floyd Curl Drive, San Antonio, TX 78229-3900, USA; 3 Department of Developmental Dentistry, School of Dentistry, University of Texas Health San Antonio, 7703 Floyd Curl Drive, San Antonio, TX 78229-3900, USA

**Keywords:** CPP-ACP, calcium phosphate, dentifrice, fluoride, hydroxyapatite, molar incisor hypomineralization (MIH), remineralization, sensitivity, toothpaste

## Abstract

Molar incisor hypomineralization (MIH) is a highly prevalent dental developmental disorder with a significant health burden for patients and high treatment needs, yet no comprehensive review article on all remineralization systems as a non-invasive treatment approach for MIH has been published. Typical characteristics of MIH-affected teeth are a lower mineral density and lower hardness compared to healthy teeth leading to sensitivity and loss of function. Thus, the use of formulations with calcium phosphates to remineralize MIH-affected teeth is reasonable. This review presents an up-to-date overview of remineralization studies focusing on active ingredients investigated for remineralization of MIH, i.e., casein phosphopeptide amorphous calcium phosphate (CPP-ACP), casein phosphopeptide amorphous calcium fluoride phosphate (CPP-ACFP), hydroxyapatite, calcium glycerophosphate, self-assembling peptide, and fluoride. Overall, 19 studies (in vitro, in situ, and in vivo) were found. Furthermore, an additional search for studies focusing on using toothpaste/dentifrices for MIH management resulted in six studies, where three studies were on remineralization and three on reduction of sensitivity. Overall, the studies analyzed in this review showed that MIH-affected teeth could be remineralized using calcium phosphate-based approaches. In conclusion, calcium phosphates like CPP-ACP, calcium glycerophosphate, and hydroxyapatite can be used to remineralize MIH-affected teeth. In addition to MIH-remineralization, CPP-ACP and hydroxyapatite also offer relief from MIH-associated tooth sensitivity.

## 1. Introduction

**Figure 1:**
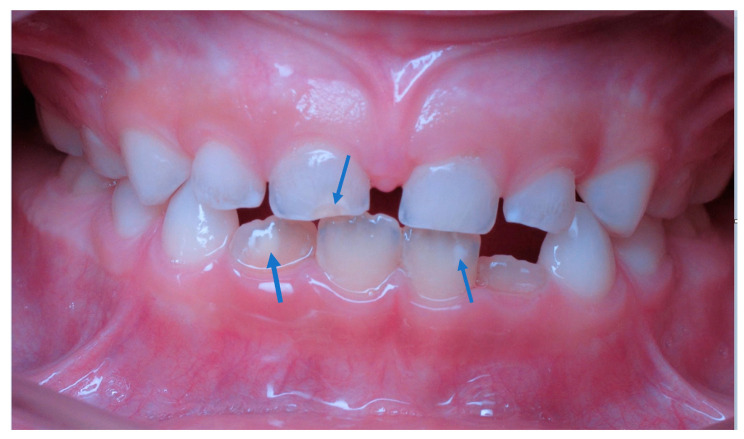
A clinical photo of molar incisor hypomineralization (MIH) showing a mild level of MIH on primary tooth #E and permanent tooth #24 & #26 (American nomenclatures).

Molar incisor hypomineralization (MIH) is a highly prevalent dental developmental disorder with a significant health burden for patients [1]. Studies reported an average global prevalence of about 14.2%, ranging between approximately 11–18% depending on the geographic region [2]. MIH is a qualitative developmental enamel defect of systemic origin, affecting at least one of the four first permanent molars, and is often associated with opacities that form on the permanent maxillary incisors or less commonly on the permanent mandibular incisors [3,4]. A clinical photo of MIH is presented in Figure 1.

Clinically, MIH is diagnosed when the opacity presented as a well-defined alteration of the translucency of the tooth enamel, was larger than 1 mm, had a color varying from creamy white to yellow to brown and was located on smooth buccal or lingual surfaces of a permanent first molar or incisor [4,5]. The etiology for the development of MIH is still unknown; however, MIH is recognized as a multifactorial dental developmental disorder resulting, e.g., from genetic as well as systemic factors. Perinatal and postnatal factors appear to be more strongly associated with the appearance of MIH than prenatal factors [6]. A recent paper describes a possible explanation involving the presence of serum albumin in the enamel matrix, inhibiting the mineralization process (“mineral poisoning”) during the enamel maturation phase [7]. While dental fluorosis and MIH can be clinically differentiated, Fernandes et al. have shown that there is likely a positive association between the severity of MIH and dental fluorosis [8]. Dental fluorosis can also be found on molars and incisors in the permanent dentition when children from birth to 6 years have a high fluoride intake (in general, all teeth can be affected by dental fluorosis) [9]. The structure, composition, and mechanical properties of MIH teeth have been analyzed using different analytical methods [10,11,12,13,14]. Compared to healthy tooth enamel, the hypomineralized enamel is poor in mineral content, lacks organization, and has widened prism sheaths and reduced calcium content but higher carbon and protein contents [12,15]. This leads to a more porous surface with reduced hardness compared to healthy teeth [10,11,12,13,14]. Consequently, susceptibility to dental caries is increased [7,16,17], and tooth sensitivity is a common problem in MIH patients [18,19,20,21]. Patients diagnosed with MIH need special dental treatment considerations and an oral care regime focused on prevention. Recommendations for the routine use of topical remineralization agents, such as fluorides, have been published [5], but the overall evidence of different treatment protocols for this patient group is low [22]. The rationale for using remineralization techniques is to increase the mineral content of the hypomineralized dental tissues in order to improve their physical properties and subsequently enhance their resistance to breakdown and caries development [23]. Thus, there is a need for new agents and more research for the oral care of MIH patients.

Calcium phosphates like casein phosphopeptide amorphous calcium phosphate (CPP-ACP; ACP: Ca*_x_*(PO_4_)*_Y_* · *n* H_2_O) and hydroxyapatite (HAP; Ca_5_(PO_4_)_3_(OH)) have gained increased attention in recent years as biomimetic/bionic ingredients, that can be used for the remineralization of enamel and dentin and for caries prevention [24,25,26,27,28,29,30] as well as for biofilm control [31,32], periodontal health [33] and whitening [34]. Hydroxyapatite particles have been shown to form mineral-mineral bridges with the natural enamel surface [35].

Since MIH teeth have a reduced hydroxyapatite content and density compared to healthy teeth [10], the use of remineralizing oral care formulations with calcium phosphates is reasonable [25,28]. Additionally, calcium phosphates (e.g., hydroxyapatite) have been shown to be beneficial for pain relief of MIH-associated tooth sensitivity [18,20,36]. Unlike fluorides which can cause dental fluorosis and other side effects [37,38,39], calcium phosphates show excellent biocompatibility and are safe if swallowed [40]. Note that nanoparticular amorphous calcium phosphate is present, e.g., in human breast milk [40].

The aim of this narrative review article was to provide an up-to-date overview of studies that investigated the remineralization potential of various active ingredients for teeth with MIH. We expect our review to generate recommendations for daily oral care for patients with MIH-affected teeth.

## 2. Search Strategy

The aim of the first search was to find all studies in the field of MIH and remineralization. Search terms were (MIH OR “molar incisor hypomineralization” OR “molar incisor hypomineralisation”) AND (remineralization OR remineralisation OR mineralization OR mineralisation) using PubMed, Google Scholar, and SciFinder. After screening both publication titles and abstracts, articles that did not focus on remineralization, as well as review articles, were excluded. Note that all active ingredients in the field of MIH-remineralization were included. Studies that were found in two or more databases were just counted as one.

The aim of an additional search was to find all studies in the field of MIH and toothpaste. For that, the following search terms were used (MIH OR “molar incisor hypomineralization” OR “molar incisor hypomineralisation”) AND (toothpaste OR dentifrice) using PubMed. After screening both publication titles and abstracts, articles that did not focus on toothpaste/dentifrices as well as review articles were excluded. All study types (in vitro, in situ and in vivo) were included. All results until 20 December 2022 were included.

## 3. Results

The results are divided into a general part (MIH-remineralization studies in general) and a toothpaste-specific part (toothpaste studies in the field of MIH).

Nineteen studies (in vitro, in situ, and in vivo) were found analyzing different remineralization strategies for teeth with MIH (Table 1). Most studies analyzed the remineralization effect of formulations with different calcium phosphates, i.e., CPP-ACP (casein phosphopeptide amorphous calcium phosphate) (10), CPP-ACFP (casein phosphopeptide amorphous calcium fluoride phosphate) (6), hydroxyapatite (3), and calcium glycerophosphate (2). One study used a self-assembling peptide. Formulations with fluoride as an active agent, i.e., not as CPP-ACFP, were analyzed in 6 studies (in most cases as fluoride varnish).

An additional search for studies focusing on using toothpaste in the field of MIH resulted in six studies, whereas three studies were on remineralization and three were on the reduction of sensitivity (Table 2).

In the field of toothpaste, a direct comparison of the MIH-remineralization efficacy and sensitivity relief between fluoride-free hydroxyapatite toothpastes and fluoride toothpastes has been published (see summaries of results in Table 3 and Table 4) [18,25]. Amaechi et al. [22] have shown in an in situ study that a hydroxyapatite toothpaste showed a practically significantly higher percentage of remineralization compared with toothpaste with 1450 ppm fluoride [25]. The mineral density measurements in this study were performed with microcomputed tomography [25] (Table 3). Ehlers et al. have shown in a randomized controlled trial with MIH patients that the group that used hydroxyapatite toothpaste tended to be less sensitive compared with the group using toothpaste with 1400 ppm fluoride [18] (Table 4). The pain sensation in response to tactile stimulus in this study was measured using the Wong-Baker FACES Pain Rating Scale from 0 (no hurt) to 10 (hurts worst) [18].

## 4. Discussion

Molar incisor hypomineralization (MIH) is a highly prevalent dental developmental disorder with a significant health burden for patients and high treatment needs, yet no comprehensive review article on all remineralization systems as a non-invasive treatment approach for MIH has been published. MIH-affected teeth are characterized by a lower mineral density and lower hardness compared to healthy teeth; thus, treatment of MIH-affected teeth with any therapeutic agent that can promote its remineralization is reasonable. Most studies in this area have been published in recent years, and the “oldest” study is from 2011 (Table 1), which clearly shows the increased research interest in this area. The presented studies show that different types of calcium phosphates can remineralize teeth with MIH defects (Table 1). These ingredients induce remineralization by providing an environment that is supersaturated in calcium and phosphate ions at the enamel surface, which stimulates crystal growth [57]. Besides remineralization, a further advantage of calcium phosphates is the reduction in MIH-associated tooth sensitivity [36]. This has been shown in studies by Ehlers et al. (fluoride-free toothpaste with hydroxyapatite) [18] and Pasini et al. (tooth mousse with CPP-ACP) [20]. Calcium phosphates, in general, can be incorporated into various oral care products such as toothpaste [25,26,58], mouthwashes [31,32], and oral gels [59]. A clear advantage of calcium phosphates is their biomimetic characteristic [40]. Consequently, higher effective doses can be used with no risk of toxicity (unlike fluoride) [29]. Furthermore, all calcium phosphates are safe for all age groups, including children, because if accidentally swallowed, they are dissolved in the stomach, just releasing harmless calcium and phosphate ions [40]. Most oral care products using calcium phosphates are based on either CPP-ACP or HAP. A limitation of the use of CPP-ACP, however, is that it cannot be used in patients with allergies to milk proteins (ACP is stabilized by casein phosphopeptides (CPP), which are proteins derived from cow milk) [5,60]. Moreover, CPP-ACP is used in form of a “tooth mousse”, in addition to the toothpaste use. In contrast, HAP can be incorporated in oral gels, used in addition to tooth brushing [61,62,63], just as it can be incorporated in regular toothpastes [18,25,29].

A comparison between the studies presented in Table 1 is difficult because many different techniques were used to measure the remineralizing effect on MIH-teeth, e.g., microcomputed tomography [25], transverse microradiography [23], microhardness test (Vickers) [42], laser fluorescence [46,47,48], Raman microscopy [43], energy dispersive spectroscopy [44], or scanning electron microscopy [28]. In most clinical studies, laser fluorescence was used to measure the in vivo remineralization effect (Table 1). Thus, for future studies using comparable analytical methods would be helpful to make results more comparable.

Despite the high prevalence of MIH [2] as well as the established importance of prevention [64] in reducing its burden, only 19 studies were found evaluating the remineralization of MIH lesions (for all active ingredients used in oral care, including fluorides). Furthermore, only 6 studies were found (three remineralization studies and three studies on the reduction of MIH-sensitivity) with a focus on toothpaste in the field of MIH (note that this search was not limited to remineralization studies) (Table 2). This is surprising since using toothpaste is one of the most important preventive measures in oral care at home [65,66]. Thus, there is a need for more research and studies, especially clinical studies, in this field. This is also in line with a recent review published by Gevert et al., who analyzed various treatment options for MIH (including at-home treatment and in-office treatment) and concluded that there is only limited evidence supporting available treatment modalities [22]. A challenge for clinical studies in the field of MIH, however, is the analysis of the efficiency of remineralization under in vivo conditions since high-resolution techniques like scanning electron microscopy or transverse microradiography cannot be performed in vivo. Consequently, in situ studies provide a promising study design to analyze the remineralization effects of oral care products [25,44]. In situ studies involves wearing intra-oral appliances bearing MIH-affected enamel blocks by human subjects during routine use of the test product. At the end of the study, the enamel blocks are harvested from the appliance and analyzed with high-resolution techniques like microcomputed tomography outside the oral cavity [25]. In general, combining in vivo, in situ, and in vitro studies represents the most promising approach for future MIH oral care research.

Notable, although fluorides are frequently recommended for MIH patients [5], the clinical evidence for using fluoride for remineralization of MIH is very low, with just a few studies investigating fluoride formulations, and these studies focused mainly on the application of fluoride varnishes [25,44,48,49,54,55] (see also Table 1 and Table 2). Restrepo et al., for example, demonstrated that the use of four applications of 5% sodium fluoride varnish did not lead to significant remineralization compared to usual home care [55].

The clinical in situ study shown in Table 3 demonstrated that regular use of hydroxyapatite toothpaste for 14 days resulted in significant remineralization of the MIH lesions [22]. This indicates that when used as a routine oral hygiene regimen by patients affected by MIH, it would serve as an effective therapy to remineralize any MIH-affected tooth. It is envisioned that supplementing this toothpaste regimen with hydroxyapatite gel or mouthwash may enhance the remineralization process. Since MIH-affected teeth have lower mineral density compared to healthy teeth, they are consequently more prone to mechanical abrasion; thus, brushing teeth with a toothbrush with soft bristles may be recommended. Additionally, whitening toothpaste with coarse abrasives tailored for stain removal may need to be avoided to minimize tooth wear.

All the findings in this review may be a knowledge base for future research in the field of MIH. As evident in this review, the research in preventive oral health care for MIH-affected patients is still limited. Overall, compared to other approaches for daily oral care, the number of published studies, as shown in Table 1 and Table 2, and consequently the clinical evidence on different calcium phosphates used for the remineralization of MIH-affected teeth, is higher.

## 5. Conclusions

Molar incisor hypomineralization (MIH) is a global challenge with a significant health burden for patients and high treatment needs. Consequently, research interest has increased, especially in recent years. Patients with MIH have special needs for their daily oral care because MIH teeth have a lower mineral density compared to healthy teeth, and the affected teeth are prone to caries, easily breakdown, and are sensitive.

The present review indicates that products for daily oral care, such as toothpastes, mouthwashes, and oral gels based on calcium phosphates, such as calcium glycerophosphate, casein phosphopeptide amorphous calcium phosphate (CPP-ACP), and hydroxyapatite can be used for the remineralization of MIH-affected teeth. With respect to MIH-remineralization, just a very limited number of studies using fluorides have been published, mainly testing fluoride varnishes (i.e., having much higher fluoride concentrations compared to regular fluoride toothpaste). In addition to MIH-remineralization with calcium phosphates, CPP-ACP and hydroxyapatite also offer relief from MIH-associated tooth sensitivity. All calcium phosphates are safe and can be used for daily oral care by MIH patients of all age groups. In contrast to CPP-ACP, hydroxyapatite can also be incorporated into toothpastes. Thus, hydroxyapatite-based products are ideally suited for the daily oral care of MIH patients.

## Figures and Tables

**Table 1:** Overview of studies (in vitro, in situ, in vivo) analyzing the MIH-remineralization effect of different formulations and active ingredients. For experimental details on in situ studies in the field of MIH, see [25]. Ordering of studies: in vitro, in situ, in vivo.

No.	Paper (Year of Publication)	Condition	Tested Products and Controls	Conclusion of the Paper Abstract
1	Evaluation of the efficacy of CPP-ACP remineralizing mousse in molar-incisor hypomineralized teeth using polarized Raman and scanning electron microscopy: An in vitro study (2022) [41]	Remineralization (Raman microscopy and scanning electron microscopy) /in vitro	CPP-ACP tooth mousse	In conclusion, there was an improvement in mineral density and organization of the hypomineralized enamel after treatment with CPP-ACP tooth mousse.
2	Evaluation of the efficacy of CPP-ACP remineralizing mousse in MIH teeth with white and yellow opacities-in vitro Vickers microhardness analysis (2022) [42]	Remineralization (Vickers microhardness) /in vitro	CPP-ACP mousse	Topical application of CPP-ACP showed an increase in the physical strength of the hypomineralized and transition areas of MIH-affected enamel, likely due to an increase in mineral content.
3	In vitro polarized Raman analysis for the evaluation of the efficacy of CPP-ACP remineralizing mousse in tooth hypomineralization (2021) [43]	Remineralization (polarized Raman microscopy) /in vitro	Casein phosphopeptide amorphous calcium phosphate (CPP-ACP)	These results allowed us to conclude that there was an improvement in mineral density and organization of the hypomineralized enamel after treatment with CPP-ACP tooth mousse.
4	Mineralisation of developmentally hypomineralised human enamel in vitro (2013) [23]	Remineralization (TMR and polarised light microscopy) /in vitro	Surface layer removal ± NaOCl pre-treatment and 14-day exposure to a CPP-ACFP solution	Lesions were highly variable, but treatment with the remineralizing solution increased mineral content (1828 ± 461 vol% min · µm, %R = 17.7 ± 5.7) and porosity decreased, demonstrating the proof of concept that the mineral content of developmentally hypomineralized enamel can be improved after eruption.
5	Remineralization of molar incisor hypomineralization (MIH) with a hydroxyapatite toothpaste: an in-situ study (2022) [25]	Remineralization (microcomputed tomography) /in situ	Hydroxyapatite-toothpaste (20% hydroxyapatite) Fluoride toothpaste (1450 ppm fluoride toothpaste)	The tested toothpaste based on hydroxyapatite can remineralize MIH lesions. Pre-treating the tooth surface with acid-etchant enhanced remineralization.
6	An evaluation of remineralised MIH using CPP-ACP and fluoride varnish: An in-situ and in-vitro study (2022) [44]	Remineralization (energy-dispersive spectroscopy: calcium and phosphorus content) /in situ and in vitro	Casein phosphopeptide-amorphous calcium phosphate (CPP-ACP)-based cream Fluoride varnish	Remineralization can be achieved in MIH-affected teeth with the use of remineralizing agents.
7	Biomimetic hydroxyapatite paste for molar-incisor hypomineralization: A randomized clinical trial (2022) [45]	Various parameters: Plaque Control Record (PCR), Bleeding Index (BI), MIH Treatment Need Index (MIH-TNI), and Schiff Air Index (SAI) /in vivo	Zinc-hydroxyapatite-based paste	Biomimetic zinc-hydroxyapatite showed a desensitizing effect when used to treat MIH.
5	Effect of remineralization agents on molar-incisor hypomineralization-affected incisors: A randomized controlled clinical trial (2022) [46]	Remineralization (laser fluorescence) /in vivo	Calcium glycerophosphate (CaGP) Casein phosphopeptide amorphous calcium fluoride phosphate (CPP-ACFP) Control (1450 ppm fluoride toothpaste)	The additional use of both mineral-containing agents in MIH-affected teeth improved these hypomineralized lesions with mineral deposition. Even if both agents could be used in the hypomineralized teeth with demarcated opacities, future studies are recommended on the long-term effect of these mineral-containing agents with longer observation and larger sample size.
6	Effect of casein phosphopeptide amorphous calcium fluoride phosphate and calcium glycerophosphate on incisors with molar-incisor hypomineralization: A cross-over, randomized clinical trial (2022) [47]	Remineralization (laser fluorescence) /in vivo	Casein phosphopeptide amorphous calcium fluoride phosphate (CPP-ACFP) Calcium glycerophosphate (CaGP)	The primary outcome was CPP-ACFP and CaGP had a positive effect in decreasing hypomineralization on MIH-affected enamel for three months period.
7	Effects of different remineralization agents on MIH defects: a randomized clinical study (2022) [48]	Remineralization (ICDAS and laser fluorescence) /in vivo	Control (oral hygiene motivation only) Fluoride varnish Paste containing CPP-ACP Paste containing CPP-ACPF	Pastes containing calcium and phosphate may be recommended for the longer-term preservation of teeth with yellow-brown defects, which showed a post-eruptive breakdown in a shorter time.
10	A comparative evaluation of CPP-ACP cream and fluoride varnish in remineralization of MIH-affected teeth using laser fluorescence (2021) [49]	Remineralization (laser fluorescence) /in vivo	Professional application of fluoride varnish Daily single application of CPP-ACP cream	Both CPP-ACP cream and fluoride varnish are equally effective in achieving remineralization of MIH-affected teeth.
11	Assessment of remineralization of hypomineralized enamel lesions using self-assembling peptide using laser fluorescence- a pilot study (2021) [50]	Remineralization (laser fluorescence) /in vivo	Self-assembling peptide (SAP)	Thus, it can be concluded that the application of SAP could use as a viable treatment option.
12	Management of a hypomineralisation of the enamel by applying a remineraliser based on zinc hydroxyapatite (microRepair) (2021) [51]	Remineralization (photo) /in vivo	Mousse based on biomimetic nanohydroxyapatite	One year after the diagnosis, all the elements involved no longer showed any symptoms.
13	In vivo comparative evaluation of esthetics after microabrasion and microabrasion followed by casein phosphopeptide-amorphous calcium fluoride phosphate on molar incisor hypomineralization-affected incisors (2019) [52]	Tooth color (photographic evaluation) /in vivo	Microabrasion Microabrasion followed by CPP-amorphous calcium fluoride phosphate (ACFP)	Microabrasion followed by the remineralizing agent can improve the aesthetics of white tooth discoloration with time.
14	The effect of casein phosphopeptide-amorphous calcium phosphate on molar-incisor hypomineralisation: A pilot study (2017) [53]	Remineralization (laser fluorescence) /in vivo	Paste containing 10% CPP-ACP Paste containing 10% CPP-ACP with 0.2% NaF (CPP-ACFP)	This pilot study shows that using CPP-ACP and CPP-ACFP had a positive effect in reducing hypomineralisation on enamel surfaces of MIH-diagnosed teeth for a one-month period. It is important to diagnose molar-incisor hypomineralisation at an early stage to prevent excessive caries development. Therefore, further clinical studies are necessary on the long-term application of these kinds of nanocomplexes.
15	Comparison of mineral density in molar incisor hypomineralization applying fluoride varnishes and casein phosphopeptide-amorphous calcium phosphate (2017) [54]	Remineralization (laser fluorescence) /in vivo	5% sodium fluoride varnish (Duraphat^®^) 5% sodium fluoride varnish with tricalcium phosphate (Clinpro^®^) Casein phosphopeptide-amorphous calcium phosphate (Recaldent^®^)	The results obtained under the conditions used here allow concluding that Clinpro^®^ was more effective in mild lesions, whereas Duraphat^®^ was more effective in moderate lesions.
16	Effect of fluoride varnish on enamel remineralization in anterior teeth with molar incisor hypomineralization (2016) [55]	Remineralization (quantitative light-induced fluorescence) /in vivo	Four applications of 5% NaF varnish, with a 1-week interval Usual home care- control	We observed no favorable effect on the remineralization of MIH lesions in anterior teeth after four applications of fluoride varnish.
18	An innovative approach to treat incisors hypomineralization (MIH): A combined use of casein phosphopeptide-amorphous calcium phosphate and hydrogen peroxide-a case report (2012) [56]	Aesthetic appearance (photographic evaluation) /in vivo	Combined use of CPP-ACP mousse and hydrogen peroxide gel	At the end of this 5-month treatment, a noticeable aesthetic improvement of the opacities was observed.
19	MIH supplementation strategies: prospective clinical and laboratory trial (2011) [28]	Mineralization, morphology, and porosity (SEM, ESEM/EDX) /in vivo	Calcium-phosphate casein	The hypothesis tested was rejected since calcium-phosphate casein improved enamel morphology in vivo.

**Table 2:** Overview of studies (in situ and in vivo) focusing on toothpaste in the field of MIH (note that the search was not limited to remineralization studies). For experimental details on in situ studies in the field of MIH, see [25].

No.	Paper (Year of Publication)	Condition	Tested Products and Controls	Conclusion of the Paper Abstract
1	Remineralization of molar incisor hypomineralization (MIH) with a hydroxyapatite toothpaste: an in-situ study (2022) [25]	Remineralization (microcomputed tomography) /in situ	Hydroxyapatite-toothpaste (20% hydroxyapatite) Fluoride toothpaste (1450 ppm fluoride toothpaste)	The tested toothpaste based on hydroxyapatite can remineralize MIH lesions. Pre-treating the tooth surface with acid-etchant enhanced remineralization.
2	Effect of remineralization agents on molar-incisor hypomineralization-affected incisors: A randomized controlled clinical trial (2022) [46]	Remineralization (laser fluorescence) /in vivo	Calcium glycerophosphate (CaGP) Casein phosphopeptide amorphous calcium fluoride phosphate (CPP-ACFP) Control (1450 ppm fluoride toothpaste)	The additional use of both mineral-containing agents in MIH-affected teeth improved these hypomineralized lesions with mineral deposition. Even if both agents could be used in the hypomineralized teeth with demarcated opacities, future studies are recommended on the long-term effect of these mineral-containing agents with longer observation and larger sample sizes.
3	Efficacy of a toothpaste based on microcrystalline hydroxyapatite on children with hypersensitivity caused by MIH: A randomised controlled trial (2021) [18]	Sensitivity (pain sensation in response to tactile stimulus (Wong-Baker FACES Pain Rating Scale)) /in vivo	HAP-toothpaste (10% HAP) Fluoride toothpaste (1400 ppm fluoride as amine fluoride)	Overall, non-inferiority in hypersensitivity relief of a toothpaste containing hydroxyapatite compared to amine fluoride could not be shown. However, the hydroxyapatite group tended to be less hypersensitive in both populations. Attrition of the PP population due to the COVID-19 pandemic led to the loss of statistical power.
4	Molar incisor hypomineralization treatment with casein phosphopeptide and amorphous calcium phosphate in children (2018) [20]	Sensitivity (to mechanical and thermal stimuli) /in vivo	Tooth mousse with CPP-ACP Fluoride toothpaste	The use of the remineralizing agent containing CPP-ACP resulted in a significant improvement in dental sensitivity in patients with MIH.
5	Efficacy of desensitizing products containing 8% arginine and calcium carbonate for hypersensitivity relief in MIH-affected molars: an 8-week clinical study (2017) [21]	Sensitivity (to evaporative (air) stimuli and tactile stimuli) /in vivo	Each child received a single in-office treatment with a desensitizing paste containing 8% arginine and calcium carbonate, followed by 8 weeks of brushing twice daily with a desensitizing toothpaste containing 8% arginine, calcium carbonate with 1450 ppm fluoride, using a sensitive toothbrush. Additionally, the corresponding mouthwash was used.	In conclusion, 8% arginine and calcium carbonate were able to reduce hypersensitivity successfully during this 8-week trial.
6	Effect of fluoride varnish on enamel remineralization in anterior teeth with molar incisor hypomineralization (2016) [55]	Remineralization (quantitative light-induced fluorescence) /in vivo	Four applications of 5% NaF varnish, with a 1-week interval Usual home care- control	We observed no favorable effect on the remineralization of MIH lesions in anterior teeth after four applications of fluoride varnish.

**Table 3:** Summary of results of a randomized, double-blind, crossover, in situ study comparing the MIH-remineralization efficacy of a fluoride-free hydroxyapatite toothpaste compared to a toothpaste with 1450 ppm fluoride [25]. The mineral density measurements were performed with microcomputed tomography. The hydroxyapatite toothpaste showed a practically significantly higher percentage of remineralization when compared with the fluoride toothpaste (percentage remineralization; mean ± standard deviation) [25].

Outcome	Hydroxyapatite Toothpaste	Fluoride Toothpaste
Combined data	26.02 ± 20.68	14.64 ± 9.60
Etched	29.26 ± 22.99	16.83 ± 9.97
Unetched	16.62 ± 5.74	10.62 ± 8.13

**Table 4:** Summary of results of a randomized control trial testing the efficacy of a hydroxyapatite toothpaste on children with sensitivity caused by MIH compared to a toothpaste with 1400 ppm fluoride [18]. The pain sensation in response to tactile stimuli was measured using the Wong-Baker FACES Pain Rating Scale from 0 (no hurt) to 10 (hurts worst). The group that used hydroxyapatite toothpaste tended to be less sensitive compared with the group using fluoride toothpaste [18].

Toothpaste	ITT Population	PP Population
Hydroxyapatite	Mean: 2.6 [95%CI]: 1.5–3.7	Mean: 2.6 [95%CI]: 0.9–4.3
Fluoride	Mean: 3.4 [95%CI]: 2.4–4.4	Mean: 3.1 [95%CI]: 1.7–4.5

ITT: intention-to-treat; PP: per protocol; CI: confidence interval.

## Data Availability

Not applicable.
